# Spillover Effects From Next Generation EU

**DOI:** 10.1007/s10272-020-0923-z

**Published:** 2020-10-24

**Authors:** Oliver Picek

**Affiliations:** Momentum-Institut, Märzstraße 42/1, 1150 Wien, Austria

## Abstract

In July 2020, the European Commission announced its €750 billion package to revive the postpandemic European economy, Next Generation EU. The programme comprises a number of loans and grants that will be funded by taking out European debt. Although the rules on liability sharing for Next Generation EU prevent a significant mutualisation of the debt, European leaders have taken the long-recognised significant first step towards European financial and political unification that stands in stark contrast to the misguided austerity programmes during the European sovereign debt crisis.

Next Generation EU is the European Commission's €750 billion package to revive the European economy after COVID-19 that is funded by taking out European debt. In haggling over payments from the European Union, member states like to count and negotiate their net payments – the difference between what is paid into common budgets and received from them. This chauvinistic view leaves out the additional induced growth effects triggered by the package both at home (domestic multipliers) and abroad (spillover effects to other EU countries). By capturing a share of economic output from Southern and Eastern European member states that receive more grants, the economies of Northern and Western Europe grow by more than the respective portion of their contributions would suggest. The coordinated fiscal impulse ensures that every country receives a sizeable boost in economic output.

## Next Generation EU

The European Commission's Next Generation EU package includes €750 billion. Although rightly criticised as not large enough to address the gravity of the economic consequences of the virus, the instrument is nevertheless the first time in recent history that the EU will directly issue a significant form of mutual debt to redistribute and stabilise the economy. In order to finance the package, the European Commission will take out loans during the budget period 2021-2027 – in addition to the regular EU budget – and repay them with member states' contributions to low interest payments (from 2021 onwards) and sizeable principal amounts (not before the next budget period starting in 2028, and no later than 2058). Some commentators have even called this Europe's "Hamiltonian moment" reminiscent of the federalisation of American states' debt in the 18th century.

**Oliver Picek**, Momentum Institut, Vienna, Austria.

The largest and most economically significant part of the Next Generation EU package (€312.5 billion) will be paid out as grants to member states by the new European Recovery and Resilience Facility (RRF), a post-COVID-19 EU reconstruction programme. A smaller portion of the grants (€71.9 billion) will be allocated and implemented through existing EU programmes outside of the new RRF, as shown in Figure 1. Around €360 billion in loans are foreseen, which will yield useful interest rate savings on loans for member states that refinance themselves above the predicted EU bond interest rates. A small sum of guarantees completes the package.[1] A time horizon of the payments can be found in Figure 2. The largest portion of the actual payments will presumably be disbursed too late to fight the acute crisis, as around three-quarters of the RRF payouts become effective in 2023 or later (see Figure 2). Signed commitments of future payments in the RRF as well as the other grants (non-RRF, e.g. REACT-EU) are envisaged to take place from 2021 onwards in order to frontload as much as possible.

The final form of Next Generation EU, however, is not yet enacted into law. The Council will have to get approval for its proposal from the European Parliament. While the total sum of the package and the grants and loans shares are unlikely to change much, the shares for individual facilities and programmes for common European goals may increase. In any case, the plan foresees loans provided by the European Union only for Southern and Eastern EU countries, as others are assumed to be able to finance themselves at a lower or equal rate than European institutions would receive in financial markets. All countries receive grants financed by a loan

1 In particular, the European Council slashed expenses that were planned for European tasks (Just Transition Fund, InvestEU).

Figure 1
**Breakdown of Next Generation EU (€750 billion)**
2021-2027, at 2018 prices

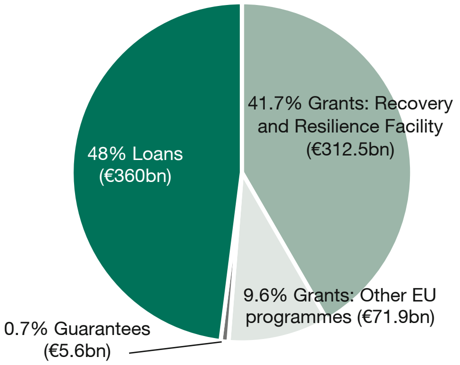

Sources: European Commission (2020a,b,c,d), Darvas (2020d), author's representation.

Figure 2
**Time horizon of Next Generation EU payments**
in billions of euros, current prices

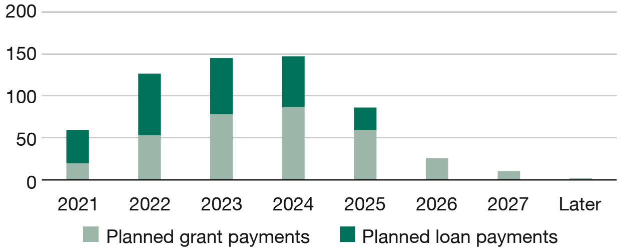

Source: European Commission (2020d), author's representation.

that is supposed to be repaid starting in 2028 and ending in 2058. As a result, the distribution of the funds cannot be considered a zero-sum game because economic stimuli can boost growth and employment for all member states while repayment occurs at a much later stage and over a longer time period.

## Distribution by country

Media, policy analysts, and informally the European Commission have provided a quite detailed preview of the distribution of the funds by country for the original proposal from May 2020 (e.g. Kafsack, 2020; Darvas 2020a,b,c), which was adjusted by the European Council (2020) on 21 July.[2] Using a set of assumptions on the eventual allocation rules per country by the Council, Darvas (2020d) has divided up the grants by amounts for individual member states, but not updated the loan amounts per country. The precise suggested distribution of the grant amounts is shown in Figure 3.[3] Loans are shown in a brighter shade above the darker grant amounts in percent of local GDP.[4]

Loans have been criticised for not being an effective way to add expenditure to the economy as they merely refinance national expenditure that would have taken place anyway – given the accommodating reaction of the European Central Bank to the crisis (Darvas, 2020c). In any case, the expected effect of loans and guarantees is unclear and difficult to estimate at this point. For the purpose of this paper, we therefore adopt the view that only grants will provide *additional* future expenditures that increase demand in the crisis-struck economies of the block. After deducting loans and guarantees from the total package sum of €750 billion, approximately €384.4 billion in grants remain. For the European Union as a whole, 3.1% of (the crisis-reduced) expected GDP in 2020 is disbursed via grants and 1.9% via loans over a six-year period.

The plan provides for a clear division of countries into those that have been hit hard by the crisis and are economically weaker (Southern and Eastern Europe), and those that can easily afford the costs of the crisis due to the unequal flow of capital within the single market, as their safe haven status allows them to draw in funds from other EU and euro area countries (Northern and Western Europe).

The bulk of spending in grants occurs only after 2022. Formulae for splitting the funds among countries include, among others, GDP per capita, the economic impact of COVID-19 (fall in GDP in 2020) and unemployment rates. They differ depending on the source of funds and concrete programmes.[5]

## The economic effects of Next Generation EU

Although a final agreement on the plan as well as some details are still missing, it is a huge political step towards sensible and joint European economic policymaking. The long-recognised need for a common fiscal response to economic crises had been acknowledged in academic and policymaking circles for a long time and became mainstream after the austerity disaster starting in 2010. This more than warrants a first analysis despite the need for a few crude assumptions.

2 Initially, the data by country was restricted to internal European Commission and member state documents, but leaked to the Frankfurter Allgemeine Zeitung (Kafsack, 2020) and later recalculated by Darvas (2020a,b,c).
3 It is presented here in percent of expected GDP for 2020 (AMECO, 15 June 2020). The amounts are converted from 2018 constant prices to 2020 constant prices.
4 For lack of a better estimate, their value is calculated indirectly by taking the total values per country of the original Commission May 2020 proposal and deducting the grant amounts of the Council July 21 proposal.
5 The implicit geographical distribution as well as the eventual share of grants, loans and guarantees is still subject to an agreement in the European Council and the European Parliament in autumn 2020.

Figure 3
**European Commission Plan: Distribution of total spending**

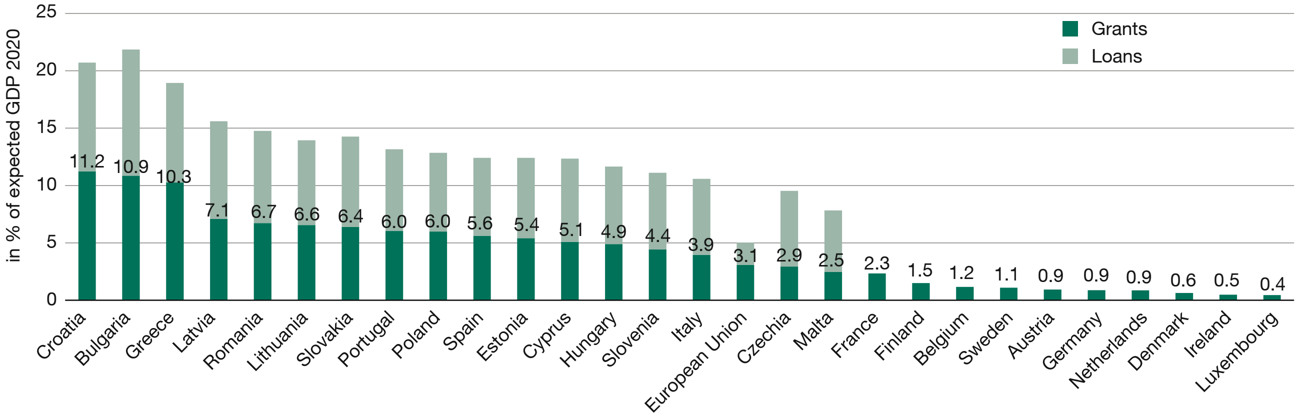

Sources: Kafsack (2020), Darvas (2020b), author's calculations.

In particular, we focus on two aspects: First, what is the increase in GDP per country due to the plan? Second, how do economic structures of member states allow them to take part in the positive effects of the plan through spillover effects from others?

## Method and data

A simple static multi-regional input-output model is employed. The analysis follows Picek and Schröder (2018, 2017), who use the World-Input Output Database (WIOD) to calculate spillover effects with an impact analysis framework (Timmer et al., 2015; Miller and Blair, 2009).[6] The cumulative future grant receipts of member states are used as a positive expenditure shock to estimate the cumulative increase of GDP. To this end, it is assumed that one-third of the EU funds will be used as government expenditure and two-thirds as investment expenditure (public and private). This is an arbitrary, yet inevitable assumption because the distribution of funds per demand category and economic sector is currently unknown. Sector allocations may be at best inferred for some parts of individual smaller programmes, but an accurate global allocation is impossible at this stage. Therefore, a second crucial assumption is made by allowing the shock to increase spending proportionally according to the current industry structure – in other words, we split each additional euro of spending (as part of the initial shock) along the industry lines that prevail according to the latest industrial (input-output) structure of the economy. The third assumption is that grants and loans have an additional effect in the sense that the expenditure would otherwise not have taken place and does not crowd out other expenditure. Fourth, there is uncertainty around the precise amount that will be spent. Implicitly, a 100% take-up rate of potential Next Generation EU funds is assumed. This may not be the case if the absorption rate of EU Structural and Cohesion funds provides any indication – the median among the member states lingers around 88% (see Figure 3 in Ionescu and Dietrich, 2015). Also, we assume that we know the precise amount that €750 billion (in 2018 constant prices) will be able to buy in the future – in other words, we assume a certain trajectory for the inflation rate and a fixed disbursement schedule over time.[7] Naturally, these assumptions introduce an unavoidable amount of uncertainty that may make the input to the simulation (the shock) slightly imprecise (Matthews, 2020). However, the qualitative results should hold provided an absence of large cross-country differences among the factors in the assumptions.

## Models of spillover effects

Three models are calculated with different multipliers to distinguish between the first-round effects arising directly from the shock and induced effects that result indirectly from increased economic activity. The direct effect of the initial expenditures plus the required intermediate input increases are depicted in the baseline model only. A Keynesian consumption multiplier based on wage income is added in the second model to arrive at an estimation of the economic effects of induced consumption. Finally, a channel that links (induced) investment spending to profits is added. Spillover effects across borders occur in all three models.

6 Building on Picek and Schröder (2018), this article uses the second version of the WIOD with the latest input-output table from 2014 and extends the analysis further.

7 According to the rules of the Multiannual Financial Framework, a 2% inflation rate for each year must be assumed to convert from constant to current prices. With the European Central Bank unable to bring inflation to 2%, however, the real value of the planned spending in current prices could be higher when inflation remains below the benchmark. Furthermore, delays or unplanned frontloading of the disbursements of funds also influence their real value.

Naturally, a note of caution is required at this point. Model uncertainty is always present and arises in input-output models mainly due to the linearity and simplicity of the model.[8] In order to make useful predictions, a crucial prerequisite for input-output models is that the economy must not be constrained by the ability of firms to supply goods and services at the prevailing prices. Therefore, the results hold less if disrupted supply lines are present in 2021 and later. On the one hand, several typical major supply restrictions such as high energy prices, hawkish central bank interest policy and shortage of labour do not constrain the economy during the current recession. On the other hand, impaired equity values in many firms may lead to a lower propensity to invest than in normal times. More important, however, is the fact that the aftermath of COVID-19 or a second outbreak thereof may significantly alter the observed consumption and investment behaviour based on historic data embedded in the model – both at the aggregate and sectoral levels. When propensities to consume and invest falter in 2020 and fail to recover entirely in 2021 and subsequent years, the multiplier implied in the model does not hold up. The growth and employment effect of the programme could then be muted to a certain extent.

Readers who are inclined to believe that the economic aftermath of COVID-19 will leave permanent marks could therefore primarily look at the results of the model with direct effects (and intermediate inputs) only, and if they do not want to rely on historic (but uncertain) consumption and investment – multipliers for the next years. Readers who believe investment will be impaired for a longer time due to the balance sheet problems of many firms, but consumption will not be (thanks to e.g. short-term work schemes), should focus on the results of the direct plus consumption-induced model. The total values for each country from the third model including direct, consumption-induced and investment-induced effects probably tell the story of an optimistic future and strong investment dynamics within a powerful rebound and upswing after the coronavirus has been safely contained.

## Main results

A note on the interpretation of the results is in order. The initial stimulus takes the form of a grant payment that takes place once between 2021 and 2027 and is not renewed or repeated. The following Figures therefore show the cumulative effect of a cumulative shock that is nonetheless a one-time event. For each country, the results are in percent of their respective GDP in 2020. However, it does not mean that real GDP will be higher by this amount at the end of a specific year or at the end of the programme period. Instead, it means that over the 2021-2027 period, the differences between a higher GDP (because of Next Generation EU) and the baseline GDP add up to the amounts shown in the graph. To receive precise yearly numbers, one would have to divide the results by the number of years under consideration weighted by a presumed disbursement schedule. The maximum effect of the programme can be expected in 2023 and 2024, when over 70% of the Resilience and Recovery Facility grants, the main part of Next Generation EU, are scheduled to be paid out. For simplicity, a division of the cumulative sum by seven (number of years from 2021 to 2027) can provide a quick estimate of the average yearly effect (in terms of GDP in 2020). Another, hypothetical, way to think about the results is the following: if all planned additional expenditures were theoretically spent in 2020 (in a non-COVID-19 economy), by how much would GDP increase in 2020? Naturally, as the one-time impulse is withdrawn in 2021 and later years, this opens up room for a subsequent (partial) fall in economic activity unless the additional income stream in 2020 has laid roots for a self-contained private sector upswing in the years following the impulse.

The main result of the present paper is shown in Figure 4 (cumulative) and Figure 5 (yearly average). The cumulative increase in real GDP over the period 2021-2027 as a result of the additional investment and government spending is presented for all EU countries in Figure 4. During the programme period, additional Greek GDP through Next Generation EU accumulates to 22.5% of GDP 2020 in the best case. And dividing over seven years, this amounts to an average yearly increase of 3.2% (in terms of 2020 constant prices as shown in Figure 5).[9] In the worst case scenario (only direct effects), without significant consumption and investment multipliers, a meagre cumulative increase of 7.7% of expected 2020 Greek GDP is recorded – or 1.1% per year. Roughly, but not precisely in line with diminishing domestic grant expenditure, the economic effect decreases successively, iterating through each Southern European country and the Central and Eastern European accession countries. The South-Eastern block is followed by France and Malta, before Germany and Austria begin to lead the geographic and political North-West flank of the Union with smaller grant amounts (and no loans), ending with Denmark. The latter face less than a 1% higher level of real GDP over the time horizon.

How do these results compare with those of other models, in particular DSGE models? European Commission staff has simulated the impact of Next Generation EU in a stylised version of their QUEST III model (see Verwey et al., 2020). While

8 Compared to DSGE or more modern macro models, however, overfitting of parameters is impossible, leaving the researcher less choice regarding the parameter input values and the subsequent results. For a discussion with more details, see Picek and Schröder (2018).

9 In the years after 2020, this increase becomes slightly smaller than 3.2% because the denominator (real GDP in future years) increases.

Figure 4

**Next Generation EU grants: Cumulative increase in real GDP 2021-2027 including spillover effects**

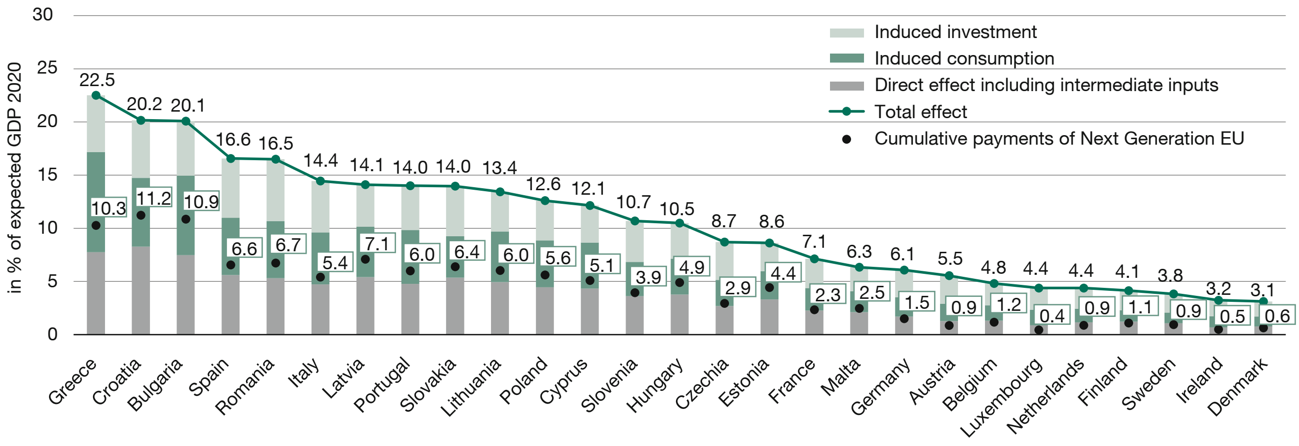

Source: WIOD, Picek and Schröder (2018), author's calculations.

Figure 5

**Next Generation EU grants: Average yearly increase in real GDP 2021-2027**

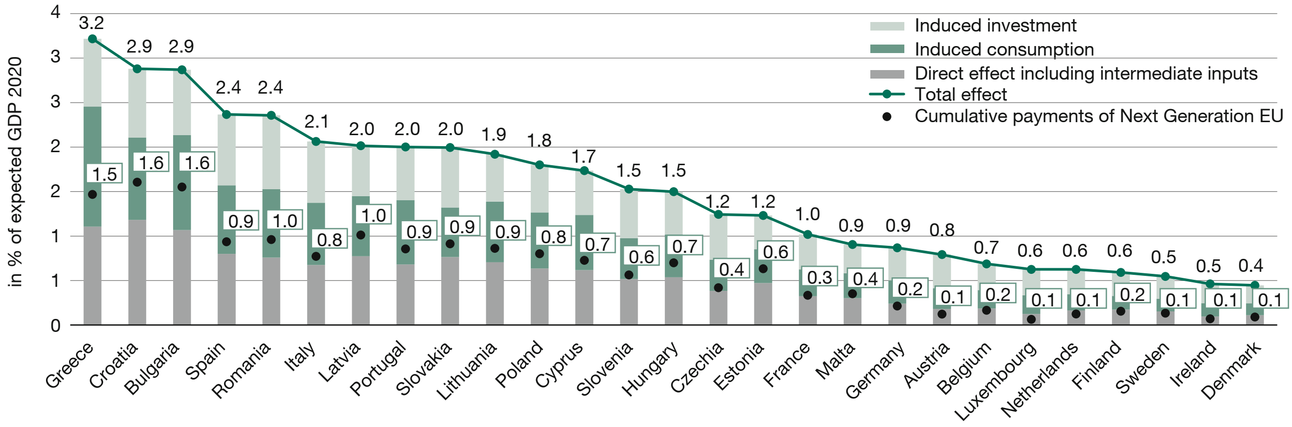

Source: WIOD, Picek and Schröder (2018), author's calculations.

not providing a detailed analysis, the article reveals that real GDP levels are 2% higher in 2024 and remain elevated by 1% until 2030 compared to a baseline scenario. Even higher income member states (in Northern and Western Europe) will experience an increase in real GDP levels of 1% in 2024. Not knowing the precise specifications of their model makes it difficult to make a sound comparison. Notwithstanding slight differences, the results of the Commission model appear nonetheless comparable in magnitude to the results presented in this paper. Among the four big euro area economies, Spain and Italy each sustain an average yearly real GDP level of over 2% above the baseline if investment and consumption follow historic trends. France sustains a yearly GDP level increase of 1% above baseline and Germany of 0.9% in the period from 2021 to 2027.

In most countries, economic effects are larger than the initial shock plus direct intermediate inputs (the first model with the smallest multipliers). However, this only applies because the simultaneous expansion in all member states 'returns' spillover effects to each country. For instance, if Austria alone were to undertake a national expenditure programme equal in magnitude to the Austrian share of Next Generation EU (0.9% of Austrian GDP in 2020), the direct effect (including intermediate inputs of the initial shock) within Austria would only yield a 0.6% increase in domestic GDP. A large part of the economic benefit dissipates – from the domestic point of view – towards foreign countries. But due to the coordinated expansion through Next Generation EU, other countries 'return the favour' and Austria manages to capture its share of their spending for an additional 1.3% of Austrian GDP – even in the most restricted model.[10] In Figure 6, the total economic effect of the plan per country is decomposed into a domestic part (green shades) and a spillover part from other countries (grey shades). The shading reflects the three models.[11] The (green) domestic part is defined as the GDP growth that results from each country running its own stimulus programme while no other country runs theirs simultaneously. This stand-alone programme is otherwise identical to the level and composition of Next Generation EU as scheduled for that country. The foreign part for each country is then simply the difference between Next Generation EU and the stand-alone expansion for that country. This spillover can be interpreted as the price each country would pay for having to go it alone when all neighbours decide to remain uncooperative and not stimulate their own economy as planned for Next Generation EU.

Despite the clear value added for all countries that a cooperative fiscal stimulus entails, significant differences prevail across member states. Taking the model with the largest effect (including consumption and investment multipliers), Greece, Croatia, Italy, Spain and Portugal rely on a domestic effect that accounts for more than 80% of the total GDP increase of the programme, owing to the fact that their domestic stimulus is large compared to other countries. On the contrary, Luxembourg, Ireland, Denmark, the Netherlands, Austria and Belgium receive more than 70% of the total effect from foreign-induced demand.

Of course, any Keynesian multiplier effect – either through consumption or investment – increases the economic benefit of the plan massively for each country. Then, even the domestic effect only leads to a higher GDP increase than the original outlays. For several countries with large initial domestic spending (South and East), the greater part of the output increase stems from domestic spending and the own multiplier effect thereof. In particular, Southern European countries – being rather closed economies in comparison – exhibit high own domestic multipliers (Picek and Schröder, 2019).

For Northern and Western countries in general, most of the total effect originates from outside the country because parts of

Figure 6

**Decomposition of cumulative GDP increases into a domestic (green) and spillover (grey) effect**

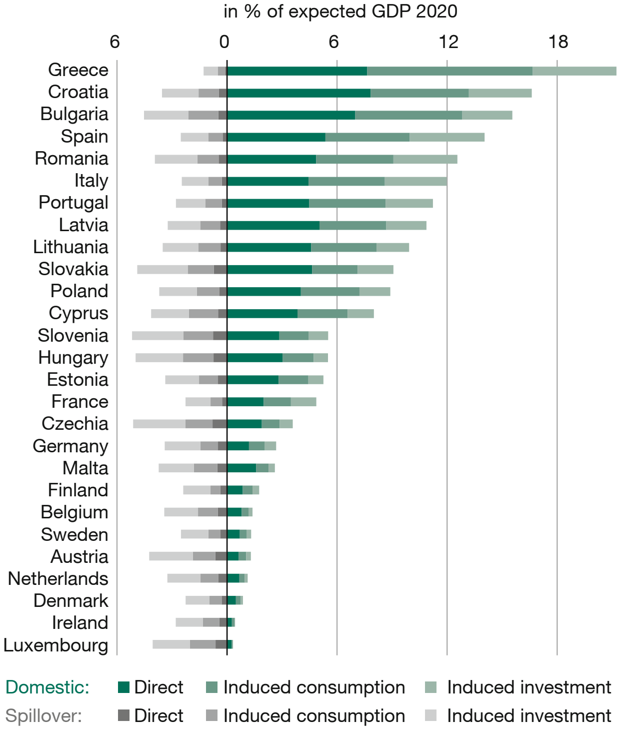

Note: The spillover effect is not negative. Both effects are positive and contribute to the total effect.

Source: Author's calculations.

the increased economic activity in foreign countries spill over. Their domestic firms and households can secure a disproportionately high share of the induced investment activity and the (resulting) consumption triggered by it. Somewhat smaller than in the investment-induced model, the induced consumption effect of grants and loans (second-round effects) increases Northern and Western EU GDPs by sizeable amounts. Of all spillover effects, the ones from the direct effect of the shocks are generally the least important for these European states.[12]

The comparatively open economies of Northern and Western Europe manage to obtain a large share of the positive economic effects of the reconstruction plan not only through the 'normal outflows' from Southern and Eastern Europe via their trade relations, but also through their favourable

10 Austria is an interesting case because it has closer geographic and economic ties to the countries of Southern and Eastern Europe than other countries from Northern and Western Europe. Before the European Council changes to Next Generation EU, made in July 2020, that gave additional grants to Germany and France, Austria was set to become the biggest beneficiary among the Northern and Western European countries, for which the plan provides only grants but no loans (right-hand section, Figure 4). There is also a political dimension: Austria nets by far the largest gain among the 'frugal four' (Denmark, Sweden, the Netherlands and Austria), making its adherence to the group relatively more costly in economic terms (Picek, 2020).

11 The three models are: direct effect, direct plus induced consumption, direct plus induced consumption and induced investment. In Figure 6, the partial effects of the three models (direct, induced consumption and induced investment) are shown just as in Figures 4 and 5.

12 To give an example using again the case of Austria: At the end of the term 2021-2027, Austria's economic output could be up to 8.8% higher than it would have been without the Next Generation EU package. Around 1.5 percentage points result from the domestic shock, while 7.3 percentage points arise from spillovers. Of the latter, 2.2 percentage points come from induced consumption and 4.1 percentage points are from induced investment (and the additional induced consumption thereof).

Figure 7

**Next Generation EU spillovers: Multiplier of the total effect relative to domestic grants**

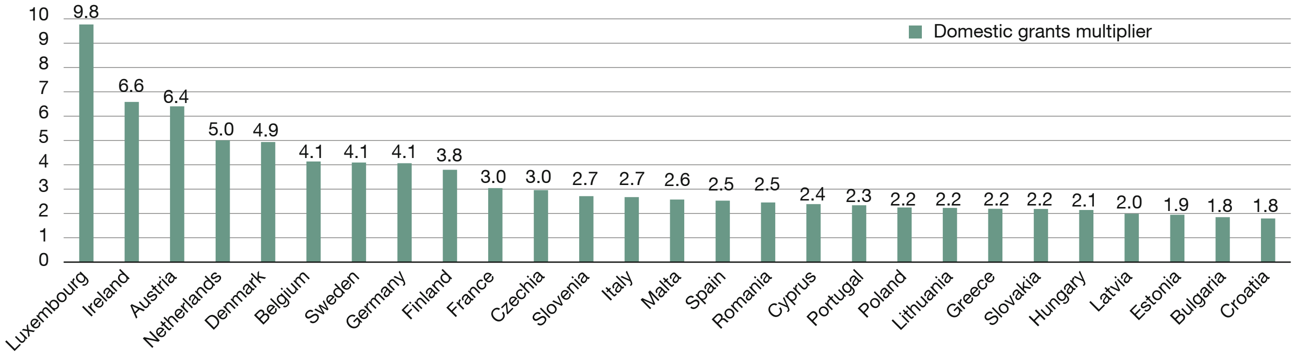

Source: WIOD, Picek and Schröder (2018), author's calculations.

industrial structure that allows them to capture demand for investment goods. This is shown in Figure 7 by relating the funds dispensed in the home country (in the form of grants) to the overall initial plus triggered GDP effects (of the whole programme). This multiplier highlights spillover effects from a clearer angle. Even disregarding the inflated value of Luxembourg due to its small size and location right between the European economic powers, Northern and Western countries (from Ireland to France) all have a much higher spillover multiplier than the Southern and Eastern countries (Czechia to Croatia).

## Conclusion

An analysis of the economic effects of the reconstruction plan shows above all the importance of a coordinated fiscal policy response to the economic consequences of the coronavirus pandemic. If only one country takes measures to stimulate the economy, part of the effect is 'lost' from a domestic perspective because this part helps foreign economies. If, however, all EU member states take measures to stimulate the economy at the same time, only a very small part is lost to other European countries outside the European Union, while the rest benefits other member states within the single market. Despite their fairly small domestic grants, even the Northern and Western European countries can thus benefit from the Next Generation EU package to a sizeable extent.
